# Best Practices and Checklist for Reviewing Artificial Intelligence-Based Medical Imaging Papers: Classification

**DOI:** 10.1007/s10278-025-01548-w

**Published:** 2025-06-04

**Authors:** Timothy L. Kline, Felipe Kitamura, Daniel Warren, Ian Pan, Amine M. Korchi, Neil Tenenholtz, Linda Moy, Judy Wawira Gichoya, Igor Santos, Kamyar Moradi, Atlas Haddadi Avval, Dana Alkhulaifat, Steven L. Blumer, Misha Ysabel Hwang, Kim-Ann Git, Abishek Shroff, Joseph Stember, Elad Walach, George Shih, Steve G. Langer

**Affiliations:** 1 Department of Radiology, Mayo Clinic, Rochester, MN USA; 2 Bunkerhill Health, Palo Alto, CA USA; 3 Universidade Federal de São Paulo, São Paulo, Brazil; 4 Carle College of Medicine University of Illinois Urbana-Champaign, Urbana, IL USA; 5 Imaging Center Onex, Groupe 3R, Geneva, Switzerland; 6 Singularity Consulting, Geneva, Switzerland; 7 Microsoft Research, Cambridge, MA USA; 8 Department of Radiology, NYU School of Medicine, New York, NY USA; 9 Department of Radiology, Emory University School of Medicine, Atlanta, GA USA; 10 Ionic Health, Porto, Portugal; 11 Johns Hopkins University, Baltimore, MD USA; 12 Department of Radiology and Biomedical Imaging, University of California, San Francisco, CA USA; 13 Children’s Hospital of Philadelphia, Philadelphia, PA USA; 14 UPMC Children’s Hospital, Pittsburgh, PA USA; 15 De La Salle University, Manila, Luzon, Philippines; 16 Hospital Selayang, Batu Caves, Selangor, Malaysia; 17 GE Healthcare, JFWTC, Bangalore, Karnataka India; 18 Department of Radiology, Neuroradiology Division Memorial Sloan Kettering Cancer Center, New York, NY USA; 19 Aidoc, Tel Aviv, Israel; 20 Department of Radiology, Weill Medical College of Cornell University, New York, NY USA

**Keywords:** Artificial Intelligence, Best practices, Checklist, Classification, Medical imaging, Paper review

## Abstract

Recent advances in Artificial Intelligence (AI) methodologies and their application to medical imaging has led to an explosion of related research programs utilizing AI to produce state-of-the-art classification performance. Ideally, research culminates in dissemination of the findings in peer-reviewed journals. To date, acceptance or rejection criteria are often subjective; however, reproducible science requires reproducible review. The Machine Learning Education Sub-Committee of the Society for Imaging Informatics in Medicine (SIIM) has identified a knowledge gap and need to establish guidelines for reviewing these studies. This present work, written from the machine learning practitioner standpoint, follows a similar approach to our previous paper related to segmentation. In this series, the committee will address best practices to follow in AI-based studies and present the required sections with examples and discussion of requirements to make the studies cohesive, reproducible, accurate, and self-contained. This entry in the series focuses on image classification. Elements like dataset curation, data pre-processing steps, reference standard identification, data partitioning, model architecture, and training are discussed. Sections are presented as in a typical manuscript. The content describes the information necessary to ensure the study is of sufficient quality for publication consideration and, compared with other checklists, provides a focused approach with application to image classification tasks. The goal of this series is to provide resources to not only help improve the review process for AI-based medical imaging papers, but to facilitate a standard for the information that should be presented within all components of the research study.

## Overview

The Machine Learning Education Subcommittee of the Society of Informatics in Medicine (SIIM) has established this primer series with the goal of providing resources for improving manuscripts published in the field of medical imaging using machine learning or deep learning techniques. The Subcommittee is made up of AI experts, Radiologists, and Imaging Informatics professionals who helped guide the development of this primer series. The vision for the series is to provide a checklist for reviewers of manuscripts to help standardize the review process and identify important components that all publications should ideally address. This primer provides an overview of the key issues in study design, data curation, and model training with a focus on algorithms that are developed for image classification tasks. This committee recently published a best practice paper on image segmentation [1]. Details from the prior work do overlap in examples not distinctly inherent to classification-type problems. We hope that this series will be useful for both the reviewers, as well as authors preparing papers in this field.

A key part of science is reproducibility. Authors must describe their methodologies and results in sufficient detail to enable readers to assess the rigor and generalizability of the work. To the extent that a work cannot be appropriately evaluated and/or reproduced, it does not advance the field. This article aims to provide a granular set of best practices to prospective authors, reviewers, and editorial boards related to publishing machine learning papers regarding medical image classification tasks. Additional entries in the series are planned that will focus on different application areas germane to medical imaging AI.

A typical classification problem uses imaging data to make a prediction. For example, the prediction might try to predict tumor type, treatment response groups, or overall survival. Our goal in this best practice paper on classification is to enhance reproducibility and replicability [2] of published manuscripts by providing a checklist to guide reviewers. Other high-level guides [3–6] have been published elsewhere and were considered when creating the current work. This current work differs from prior works in several ways. CLAIM, Tripod-AI, and CONSORT-AI function as reporting standards. They ensure transparency and promote reproducible research, but they each focus on slightly different use cases (retrospective imaging, predictive modeling in general, and clinical trials, respectively). This current work is more specialized, explicitly providing guidance on how to review classification studies. It offers a deeper dive into classification-specific metrics, study design pitfalls, and thoroughness checks aimed at the manuscript evaluation stage. We believe that the developed screening tool is unique to our checklist and will highlight areas of strengths and weaknesses of a submitted paper. Moreover, the checklist may identify specific sections where deficiencies exist across multiple manuscripts. This type of analysis will allow the development of strategies and resources to address these limitations and further promote the reproducibility of AI research in medical imaging.

This article is presented in a similar manner as a typical peer-reviewed manuscript. Each section contains suggested content, followed by an expanded description for each checklist item. We anticipate the checklist created (Table 1) will be used as a guide during manuscript preparation as well as review. In addition, authors can use the checklist to self-report when submitting their work for review.

**Table 1:** Checklist for AI-based Medical Imaging Papers: Part 1 – Classification

Section	No.	Item
Introduction	I-1	Contains sufficient background information related to relevant use case
	I-2	Discusses relevant and related work
	I-3	Details literature gap addressed by current study
	I-4	Provides study objectives
Methods	M-1	Defines dataset origin
	M-2	Provides appropriate oversight committee approvals for original data
	M-3	Outlines steps to protect patient privacy
	M-4	Specifies image acquisition parameters
	M-5	Defines and justifies reference standard
	M-6	Provides software and data annotation method, annotators’ experience
	M-7	Partitions dataset into training, validation, and test sets at patient level
	M-8	Defines and justifies pre-processing method
	M-9	Describes data augmentation strategies used
	M-10	Discusses model architecture
	M-11	Presents hyperparameter choice and training protocol
	M-12	Describes strategies for handling class imbalance
	M-13	Details core software versions and libraries
	M-14	Assesses inter/intra variabilities, if possible
	M-15	Identifies primary performance metric, explains appropriate rationale
	M-16	Describes statistical analysis methods
	M-17	Uses sensitivity analyses to test model robustness
Results	R-1	Includes STARD-like diagram
	R-2	Provides data subset clinical information in tabular form
	R-3	Provides Data Availability Statement
	R-4	Includes sufficient analysis
	R-5	Uses explainability methods to demonstrate a reasonable and/or new explanation
	R-6	Uses failure analysis with reasonable hypotheses for incorrect model predictions
Discussion	D-1	Summarizes results concisely
	D-2	Suggests clinical use cases
	D-3	Compares findings to recent, relevant studies
	D-4	Describes limitations
Conclusion	C-1	Concise
	C-2	Positions work in the context of state-of-the-art practice, if applicable
	C-3	Recommends future work, if applicable
	C-4	Agrees with reported results
Code	Co-1	Provides code or justifies its omission

## Introduction

The introduction section needs to provide the rationale as to why the study was performed and why the study question is relevant. The use case for having the particular classification and relevant quantitative information should be clearly spelled out. References to prior studies, including their successes and failures, should be comprehensive. The contributions made or knowledge gaps filled by the current study need to be clearly stated. The introduction section should not include broad generalizations or a wide scope which detracts from the purpose of the paper. The last paragraph should state the objective of the study.

### [I-1] A Sufficient Level of Background Information Related to the Image Classification Use Case Is Given

Background material may include information about the target population, prevalence, and significance of the problem being addressed. In general, these introductory paragraphs should describe how many people or systems are affected in a given region, the impact on quality of life, operational efficiency, or resource utilization (e.g., physical limitations, economic burden, need for ongoing monitoring, or delays in processing). For instance, if the goal is to classify medical images to aid in diagnosis, describe the number of organizations or patients impacted and potential consequences of misclassifications.

The background should also address currently accepted tools, techniques, or strategies used to investigate or address the issue, including clinical practices, workflow optimization methods, and data analysis frameworks. Existing gaps or inefficiencies in current approaches may be highlighted to underscore the relevance of the proposed solution in improving outcomes, reducing costs, or streamlining workflows.

### [I-2] Discussion of Relevant and Related Work Is Comprehensive

The work described should position the study within existing image classification literature. The paragraphs within the introduction should answer questions such as:

- How have others approached similar problems in the past?
- What is the current state of the art in the field?

### [I-3] Details Are Provided Regarding the Gap Filled by the Current Study

The study should clearly highlight the shortcomings in the existing image classification literature. If a new approach is described, the significant contributions it makes beyond those proposed in prior studies should be explained.

### [I-4] There Is a Clear Summary of the Study Objectives

The study’s aims and hypothesis should be clear. Studies with multiple goals should contain primary and secondary aims.

## Methods

The methods section should provide sufficient detail regarding data sources, approaches, and the software utilized.

### [M-1] Dataset Origin Is Well-Defined

The origin of the dataset needs to be clearly detailed (original and proprietary or public). The collection and data transfer steps (for example, in the case of a multi-site study) and the storage medium (on premise, cloud, internal or external) should be described. The method used to assess dataset quality should be explained (human supervised or software). Finally, the image format should be stated (DICOM, PNG, JPEG, TIFF, NIfTI, etc.). Investigators should explain the rationale behind the imaging exam type and parameters used. “The process and tools chosen to identify, query, and extract data from Electronic Medical Records/RIS/PACS should be described.” The authors should provide the date range of the dataset and the specific inclusion and exclusion criteria.

An example of suggested content covering [M-1] is as follows:

The dataset used in this study was acquired from a publicly available repository (XYZ Database) which aggregates medical imaging data from two tertiary-care hospitals (Hospital A and Hospital B). All images were retrospectively collected between January 1, 2019, and December 31, 2020. Data were transferred to a secure, on-premises server via encrypted protocols to maintain patient confidentiality. The images were stored in Digital Imaging and Communications in Medicine (DICOM) format, consistent with standard clinical practice. Prior to analysis, each DICOM was reviewed for completeness and clarity by trained radiology technicians, ensuring human-supervised quality control. Axial T1-weighted MRI scans were selected because they offer high soft-tissue contrast optimal for identifying the lesions of interest. Metadata from the hospital’s Radiology Information System (RIS) and Picture Archiving and Communication System (PACS) were used to query and extract the relevant studies, filtering out any scans with excessive motion artifacts or incomplete series. Inclusion criteria required patients to have a confirmed diagnosis and at least one high-quality axial T1-weighted series; any scans failing these criteria were excluded. This approach yielded 1,200 patient examinations, which served as the final dataset for our classification experiments.

### [M-2] If Data Are Original, the Appropriate Oversight Committee Approvals Are Detailed

Authors should report the institutional review board (IRB) approval and should state if locally applicable regulations (e.g., the health insurance portability and accountability act “HIPAA” in the USA and the general data protection regulation “GDPR” in Europe) have been respected. Information regarding informed consent should be included, along with the reason if it was waived.

An example of suggested content covering [M-2] is as follows:

This study was reviewed and approved by the Institutional Review Board (IRB) at our University, ensuring compliance with all local regulations and international standards (e.g., HIPAA for patient privacy, GDPR for data protection). Informed consent was obtained from all participants; in cases where participants could not provide consent, a waiver was granted by the IRB based on minimal risk criteria. All data were fully de-identified prior to analysis.

### [M-3] Steps to Protect Patient Privacy Should Be Outlined

Data privacy methods should be described (anonymization, pseudo-anonymization, or de-identification) and relevant technological tools or software used listed (such as encryption tools or DICOM anonymization software). This section should also include the description of advanced anonymization techniques such as defacing on head cross-sectional imaging and optical character recognition, and if human verification of each image anonymization was performed. In addition, if metadata were partially removed to retain useful information for training, the authors should describe which metadata were removed and which remain available in the dataset.

An example of suggested content covering [M-3] is as follows:

All images and associated metadata underwent de-identification using the DICOM Anonymizer before transfer to our secure server. Protected Health Information (PHI) fields were removed, and non-critical patient identifiers were replaced with pseudonyms. In head MRI datasets, defacing was performed to ensure facial features were unrecognizable. An experienced radiology technician manually verified that no visible PHI remained. Some metadata fields (e.g., scanner model) were retained for training purposes, while all patient-specific information (name, birth date, medical record number) was fully removed or encrypted.

### [M-4] Specifics Regarding Image Acquisition Parameters Are Clear

Details regarding how images were acquired and whether multiple vendors and/or institutional data were used should be clarified. State how data uniformity was established; provide the inclusion and exclusion criteria used to meet the protocol specifications. Provide details regarding the data curation process to ensure wrong images were not included.

A minimum set of information should be stated for each modality (note that this is not all-inclusive as additional modalities have unique considerations, as well):

1. Radiographs: body part and view
2. CT: body part, plane, contrast media (and bolus timing), if applicable
3. MRI: body part, plane, sequence type, contrast media (and bolus timing), if applicable

If data were acquired from multiple scanners/vendors, the range and distribution of acquisition parameters should be described. As protocol heterogeneity usually leads to more robust and generalizable models, the dataset should have diverse acquisition parameters.

An example of suggested content covering [M-4] is as follows:

All MR acquisitions were acquired at our institution on a ‘VendorName’ 3 T scanner (‘Specific scanner details’) in the supine position utilizing a multichannel surface coil. No intravenous contrast was used. The sequences included in the imaging protocol were conventional single-shot fast spin echo axial, coronal, and sagittal scout images, followed by the T1-weighted MR images used in the study (spoiled gradient sequence, with TR = 80 ms, TE = 3.2 ms, 25º flip angle, and reconstructed voxel resolution in-plane of 1.5 mm, and slice thickness of 3 mm). Images were acquired under a single breath-hold. A manual review guaranteed that sequences different from those described were not included in the dataset.

### [M-5] Reference Standard Is Clearly Defined and Justified

Details regarding the classes/labels and the classification task, in addition to how classes are defined, need to be clear. How images obtained compare to pathology, surgical findings, radiological image-based descriptions, radiological reports, and radiologist’s impressions should be clarified.

The reference standard is the reference against which the proposed method is compared. For a diagnostic test, this reference standard should be a widely accepted test or the reference standard for the diagnosis, but it can also be based on the diagnosis provided by experienced readers, especially to benchmark an algorithm designed to detect radiologic abnormalities [7, 8].

An example of suggested content covering [M-5] is as follows:

Each image was assigned to one of four diagnostic classes, defined according to the World Health Organization (WHO) guidelines for tumor staging. For surgical cases, labels were confirmed using final pathology reports; for non-surgical cases, two experienced radiologists independently reviewed the imaging and generated consensus labels based on standardized criteria. This multimodal labeling strategy served as the reference standard, ensuring that both pathological and radiological findings informed the final classification.

### [M-6] The Software and Method for Data Annotation is Clear and the Experience of the Annotator is Provided

The strategy and methods for annotating the data need to be clear with appropriate rationales explained, including.

- Markups used
- Reports used
- Level of annotation: pixel/voxel, bounding boxes, slice-level or study-level (for 3D data)
- Time tracking
- Annotation software used
- Number and experience of human annotators
- Inter/intra rater variabilities of annotations

An example of suggested content covering [M-6] is as follows:

Two board-certified radiologists with over ten years of clinical experience each used the Annotation Tool (vX.X) to delineate bounding boxes around lesions on axial slices. Radiology reports guided the annotation process, ensuring consistency with clinical practice. Time spent on each case was tracked automatically by the software. Inter-rater variability was calculated using Cohen’s kappa, yielding a value of 0.82, indicative of high agreement. Any discrepancies were resolved through consensus discussion, and all final annotations were stored in a secure format compatible with downstream analysis.

### [M-7] Dataset is Partitioned into Training, Validation, and Test Sets

The method for splitting the data into training, validation (development), and test sets must be clearly defined. It should be clear if an independent test set was used. The study dates for all datasets should be included. A flowchart showing the various datasets may be helpful.

Dataset creation methodology and strategy (e.g., single training, validation and test split, K-fold cross-validation) should be included. The size of each partition should be indicated. Explain the rationale for the dataset size including data availability and access limitations and statistical power estimation. Use of created synthetic images should be stated and methodology explained. It is important to note the importance of using K-fold cross-validation when the dataset is small, given the large variance that can occur in performance estimation with single-fold validation. Ideally, the dataset split is done at the patient level if the dataset contains more than one image from a specific patient to ensure that patients are not present across multiple partitions.

An example of suggested content covering [M-7] is as follows:

We included all images acquired between January 2019 and December 2021 and randomly divided them at the patient level into training (70%), validation (15%), and test (15%) sets to ensure no overlap of subjects across partitions. For robustness, a fivefold cross-validation procedure was also employed within the training set, mitigating high variance in performance estimates given the moderate dataset size. The final independent test set remained untouched during model development. Where data were limited, synthetic images were generated via domain-specific augmentation strategies to increase diversity. A flowchart illustrating the partitioning steps is provided in Figure X.

### [M-8] The Method of Pre-processing is Clearly Defined and Justified

Data preprocessing is critical to understand how the study can be reproduced and compared in the future. Different considerations need to be given for different modalities. For instance, CT intensities are much more standardized than those from MR. Any process that changes pixel/voxel values and numbers from the original image prior to being input to the deep learning algorithm is considered pre-processing; such image manipulations should be specifically described. Pre-processing may include changing image resolution (up-sampling or down-sampling) and re-sampling, windowing (in the case of CT), signal intensity modification and skull stripping (for MRI), cropping, standardizing using the mean and/or standard deviation of image intensities, or many other image processing techniques.

An example of suggested content covering [M-8] is as follows:

CT Hounsfield units were windowed using a window level of 50 and window width of 100 and converted to 8-bit pixel values in the range of [0, 255]. Pixel values were normalized to the range of [0, 1] and standardized using the normalized ImageNet mean and standard deviation per PyTorch convention. During both training and inference, images were padded to square and resized to 224 × 224 pixels.

### [M-9] Data Augmentation Strategies Are Described, if Used

Data augmentation methods can be used to discourage model sensitivity to known invariants and help improve its generalizability. Authors should state which methods were performed and their corresponding parameterizations (e.g., rotations of up to 15 degrees, random crops of 224 × 224 pixels with 0-padding as needed). Some common augmentation approaches may not be justifiable in medical imaging.

An example of suggested content covering [M-9] is as follows:

To improve model generalizability, we applied domain-appropriate augmentations, including small random rotations (± 10°), horizontal flips, and random crops of 224 × 224 pixels with zero-padding as needed. These choices were informed by clinical knowledge, ensuring that rotations beyond ± 15° or flips in certain planes, which could distort anatomical realism, were avoided. Parameter ranges were selected to balance augmentation diversity with clinical plausibility.

### [M-10] Discussion of the Model Architecture Is Clear

The model architecture and appropriate references should be clear. Novel architectures should include a brief summary in the methods section with full details in the appendix (architecture diagrams are encouraged for clarity of presentation). State if the model architecture was selected empirically or if there is a specific reason derived from known success of that architecture in the literature. Also, the use of pre-trained weights (or the weight initialization technique) should be mentioned.

An example of suggested content covering [M-10] is as follows:

Our model architecture is a DenseNet121 backbone with 2 fully connected layers (10 classes, sigmoid activation, dropout probability 0.5) after global average pooling. Sigmoid activation with 10 classes was used to account for the multi-class, multi-label problem, and the model was trained from scratch using He weight initialization.

### [M-11] Hyperparameter Choice and Training Protocol Are Presented

Important training and model hyperparameters (number of epochs, learning rate, model width, model depth), as well as training protocol (optimizer, loss function), are clear. If the final hyperparameters were discovered via hyperparameter optimization, the method should be detailed. Validation during training is clearly described.

An example of suggested content covering [M-11] is as follows:

Models were trained for 100 epochs using cross-entropy loss. The Adam optimizer with default parameters was used with an initial learning rate of 1.0 × 10^−3. Validation was performed every 5 epochs, and the learning rate was decreased by a factor of 10 if the validation loss did not improve after 10 epochs. Training was stopped early if the validation loss did not improve after 30 epochs.

### [M-12] Strategies for Handling Class Imbalance Are Described

Strategies for handling class imbalance (weighted loss, balanced sampling) are described, if used. The rationale of the different classes included in the dataset should be explained. If a specific dataset enrichment has been performed, it should be described and its rationale explained.

Examples of suggested content covering [M-12] include the following:

Data were sampled during training such that the proportion of each class in one epoch was the same across all classes.

Influenza viral pneumopathy, atypical bacterial pneumopathy, and cryptogenic organizing pneumonia chest CT cases have been included in the dataset because they have similar appearances to SARS-CoV2 viral pneumopathy, for which we are developing this model.

### [M-13] Core Software Versions and Libraries Are Detailed and Referenced if Necessary

An example of suggested content covering [M-13] is as follows:

Python 3.7.6 was used with PyTorch 1.4.0 and scikit-learn 0.22.

### [M-14] Assessment of Inter/Intra-rater Variabilities Is Provided, if Possible

For many studies, it is critical to know the inherent variability of the task, particularly for human readers. This gives the AI practitioner an idea of when a model is performing as well or even better than human readers. Also, it is often not possible to have this information, particularly for studies that scrape data from prior reports.

Examples of suggested content covering [M-14] include the following:

To quantify the consistency of human annotations, two board-certified radiologists independently labeled a randomly selected 10% subset of images. Inter-rater agreement was evaluated using Cohen’s kappa, resulting in a value of 0.80, while intra-rater variability was determined by having each radiologist re-label the same subset after three weeks (kappa = 0.82). These metrics provide a benchmark for comparing the model’s performance against human-level variability.

### [M-15] A Primary Performance Metric Should be Identified with Appropriate Rationale

Examples of suggested content covering [M-15] include the following:

The primary metric to evaluate model performance was the area under the receiver operating characteristic (ROC) curve, which measures the discriminatory capacity of the model.

or.

The primary metric of interest was the specificity at 95% sensitivity, as our model is intended to screen out negative studies without missing positive studies. [9, 10]

Authors should benchmark the performance of the AI model to radiology experts (when applicable). For classification tasks, include estimates of diagnostic accuracy and their precision (such as 95% confidence intervals) [9]. When the direct calculation of confidence intervals is not possible, report nonparametric estimates from bootstrap samples [10]. Authors should apply appropriate methodology such as ROC analysis and/or calibration curves. When interpreting the final model, report what variables were shown to be predictive of the response variable [10]. It is also important to note that a single metric like the area under ROC has known limitations, particularly in the context of imbalanced datasets. In such cases, metrics like the Mathews Correlation Coefficient (MCC) and precision-recall curved (PRC) provide a more accurate reflection of classification performance. In general, it is ideal that a set of metrics are presented, such as sensitivity, specificity, positive predictive value (PPV), negative predictive value (NPV), accuracy, area under ROC, and F1-score, as well as to include MCC and PRC when evaluating performance on imbalanced datasets.

### [M-16] Methods for Statistical Analysis Are Described.

Statistical analysis methodology, including presentation of the criteria for establishing statistical significance, should be provided. Authors should state how diagnostic accuracy was evaluated and whether the statistical methods were appropriate for the sample size.

An example of suggested content covering [M-16] is as follows:

Bootstrapping was used to determine 95% confidence intervals (CI) for each performance metric and to quantify the 95% CI for performance differences between radiologist and model predictions. P-values were calculated using the permutation test, p < 0.05 serving as the threshold for statistical significance.

### [M-17] Sensitivity Analyses Were Performed to Test the Model’s Robustness to Various Assumptions.

The authors should report the type of sensitivity analysis performed. Statistical methods must be appropriate for the sample size and task.

An example of suggested content covering [M-17] is as follows:

The test set was resampled at 50%, 10%, and 1% of the original positive class prevalence to evaluate robustness against possible prevalence in the real world. To assess performance on lower quality images, images in the test set were also resized to ¼ resolution and re-interpolated to their original size. Complete results are available in supplemental materials.

Authors can perform additional analyses, such as testing model performance across different subgroups (e.g., age, sex), or use alternative thresholds for classification.

## Results

The results section should provide sufficient details to assess the performance of the approach being presented. Common ways to evaluate performance include.

- ROC curves and areas under the curves, often using the DeLong method or bootstrapping for statistical comparisons
- Decision metrics such as sensitivity, specificity, positive predictive value, and F1 score, with chosen threshold

Discrimination performance does not guarantee good model output calibration (agreement between real probabilities and the predicted probabilities). Thus, we recommend the use of a calibration plot and/or the Hosmer–Lemeshow goodness-of-fit test^5^ if well-calibrated probabilities are a desired property of the model output.

### [R-1] A STARD-Like Diagram is Provided

A STARD-like diagram [9] provides details regarding the number of studies/series/images before and after inclusion/exclusion criteria.

### [R-2] A Table with the Clinical Information of Each Data Subset Is Shown.

Clinical information of each data subset is shown in table form with appropriate statistical comparison. If cross-validation was performed, clinical information can be shown for the entire dataset.

### [R-3] Data Availability Statement is Present.

Ideally, authors should try to make their datasets available. This includes the labels and the train, validation, and test set indices. In the case of institutional data, this may not be possible. Regardless, a data availability statement should be mentioned. Examples of data availability statements include the following:

“The datasets created in this study are not publicly available because <reasons>”

“This data is available at <repo link> under <license> license.”

“This study used an open-source dataset from <citation>.”

If data cannot be shared, consider providing a subset of anonymized data or simulated data that mirrors the study’s characteristics.

### [R-4] Sufficient Analysis Was Performed

Sufficient analysis is often presented in the form of reporting accuracy, sensitivity, specificity, positive predictive value, negative predictive value, F1 score, chosen threshold, area under the ROC curve, ROC curve, area under the precision-recall (PR) curve, PR curve, and Dice similarity coefficient for test sets, when appropriate.

Appropriate statistical analyses with *p* values and confidence intervals should be performed. If comparisons with radiologists are performed, they are presented as well. Calibration was assessed, though this is not always relevant; for example, it may be less important in some binary decision-making problems. Computational expenses and run-times should be presented. When performed, the hardware platform should be described in detail. Subgroup analyses and stratified performance metrics should be included to provide a detailed understanding of model behavior.

In medical imaging classification tasks, the choice of performance metrics should be guided by both clinical and data-related considerations. For instance, in contexts where missing a disease could have severe consequences, high sensitivity (recall) becomes a priority, ensuring that most positive cases are detected. On the other hand, if avoiding unnecessary procedures is critical, high specificity is emphasized to reduce false positives. Metrics like F1-score or Balanced Accuracy offer more balanced views when both classes have different prevalence rates or when you want to weigh false negatives and false positives more evenly. Meanwhile, area under the ROC gives a comprehensive view of model performance at different thresholds, but can be overly optimistic with imbalanced data, making Precision-Recall AUC a more suitable choice in such cases.

A common pitfall is relying solely on accuracy, especially in rare disease detection where high accuracy might merely reflect a model’s ability to identify the majority class (normal cases) correctly. Similarly, while area under the ROC is widely used, it can hide poor performance on minority classes. Overlooking calibration (e.g., via Brier Score) is another shortcoming, as models might calculate probabilities that are not clinically meaningful. Finally, interpreting metrics without a confusion matrix can mask the model’s specific weaknesses, like excessively high false negatives, highlighting the need to examine multiple metrics and adjust decision thresholds based on real-world clinical implications.

### [R-5] Explainability Methods Are Used to Demonstrate a Reasonable and/or New Explanation

For classification tasks, explainability methods are often presented in the form of saliency/attention maps used to show the regions within the image that activate most when making a prediction/decision by the model.

### [R-6] Failure Analysis with Reasonable Hypotheses for Incorrect Model Predictions Is Performed

Failure analysis includes performing sanity checks of incorrectly classified cases with hypotheses for failure being thoroughly described.

## Discussion

The discussion should offer a concise summary of the results section, without excessive repetition. Results should be interpreted in the context of clinical utility and suggestions made for clinical use cases. Comparisons with prior studies should be performed. Limitations, especially relating to generalizability, should not be overlooked.

### [D-1] The Results Are Summarized in a Concise and Coherent Manner

Concisely summarized results should include appropriate interpretation of image classification metrics.

### [D-2] Clinical Use Cases Are Suggested

Clinical use cases should be suggested. For example, include a detailed discussion on how the method would change clinical workflow and identify the potential benefits for the multiple stakeholders involved (patient, referring physician, radiologist, institution, etc.).

### [D-3] Proper Comparison Is Done between the Current Study and the Relevant Ones in the Recent Literature

A comparison between findings and previously published literature should include advantages and disadvantages of the novel method.

### [D-4] Limitations Are thoroughly Described

Highlighting the limitations of a study is an important component of published literature. Important components include a discussion of the settings in which the results of the study are appropriate and when they are not. For example:

- Is the test data not representative of routinely acquired data?
- Are there manual pre-processing steps that would be cumbersome to implement in a clinical workflow?

In general, authors should provide guidance on what issues the reader should be aware of when interpreting results. Example questions that need to be presented either within the discussion section or within other sections of the manuscript are:

- Was this multi-institution or single institution?
- Is the AI model generalizable to other datasets?
- What are the technical constraints in developing the current AI model?

## Conclusions

This section should offer a “takeaway message” for the reader. As such, it should focus on two areas: impact (preferably in the practical or clinical context) and unresolved questions to be addressed in further research.

### [C-1] Concise Presentation

The conclusion does not simply reiterate the sentiments provided throughout the manuscript but captures the main take away point(s) from the study.

### [C-2] Proper Positioning of This Work in the Context of State-of-the-Art Practice, If Applicable

The work presented should answer the question, “How does this work align with the current state of the field?”.

### [C-3] Recommendations for Future Work, If Applicable

Authors should propose future studies that build on the current work. Specific suggestions for next steps, such as validating the model in larger, multicenter studies; exploring other modalities; or integrating the model into clinical trials, should be provided.

### [C-4] The Conclusion is Adequately Supported by the Results of the Study.

In general, the conclusions should not be a surprise and should be well supported by the study’s results.

## Code

### [Co-1] Code Is Made Available, or If Not, is Justified Within the Manuscript as to Why It Is Omitted.

In general, a review of code could be the subject of an entire paper. However, it is important that details regarding code availability or lack thereof be clearly conveyed.
